# Supplementary material for: Social Disconnection in the Brain: Loneliness and Age across Networks using Graph Theory
Source: bioRxiv. 2026 Feb 6:2026.02.03.703621. Preprint. [Version 1] doi: 10.64898/2026.02.03.703621 (PMC12889728; doi:10.64898/2026.02.03.703621)

Supplementary Materials

To obtain positive-only connectivity weights, the negative weighted edges were omitted, and for negative-only connectivity weights, the positive weighted edges were omitted (Rubinov & Sporns, 2011). For both positive-only and negative-only graph-based measures, no association with loneliness, nor the interaction between loneliness and age was observed. Regarding positive-only graph-based measures (Table S7, Figure S1), the association between age and nodal graph-based measures were consistent with absolutized measures. Age also showed consistent positive association with the *average shortest path length* (z = 3.53, p_{FWE} = .0002). However, the association between age and *modularity* was not significant (z = -.65, p_{FWE} = .5052) (Figure S2). Regarding negative-only graph-based measures (Table S8, Figure S3), the associations between age and nodal graph-based measures were significantly attenuated. On the other hand, age was positively associated with the *average shortest path length* (z = 2.57, p_{FWE} = .0060), whereas no association with *modularity* (z = 1.64, p_{FWE} = .0822) (Figure S4).

Supplementary Reference

Rubinov, M., Sporns, O.: Weight-conserving characterization of complex functional brain networks. Neuroimage. 56, 2068–2079 (2011). https://doi.org/10.1016/J.NEUROIMAGE.2011.03.069

Glasser, M.F., Coalson, T.S., Robinson, E.C., Hacker, C.D., Harwell, J., Yacoub, E., Ugurbil, K., Andersson, J., Beckmann, C.F., Jenkinson, M., Smith, S.M., Van Essen, D.C.: A multi-modal parcellation of human cerebral cortex. Nature. 536, 171–178 (2016). https://doi.org/10.1038/nature18933

List of Supplementary Tables

Supplementary Table S1 Age and Nodal Strength Association Results

Supplementary Table S2 Age and Nodal Closeness Centrality Association Results

Supplementary Table S3 Age and Nodal Betweenness Centrality Association Results

Supplementary Table S4 Age and Nodal Eigenvector Centrality Association Results

Supplementary Table S5 Age and Nodal Clustering Coefficient Association Results

Supplementary Table S6 Age and Nodal Participation Coefficient Association Results

Supplementary Table S7 Summary of Age and Graph-Based Brain Functional Connectivity Results: Positive-Only Connectivity Weights

Supplementary Table S8 Summary of Age and Graph-Based Brain Functional Connectivity Results: Negative-Only Connectivity Weights

List of Supplementary Figures

Supplementary Figure S1 Age and Graph-Based Brain Functional Connectivity Association Maps: Positive-Only Connectivity Weights. Color bar shows the z-score from Aspin-Welch's test for using exchangeability blocks to adjusted for four scanning sites and variance was estimated for each block, implemented in FSL PALM

Supplementary Figure S2 Association of Age and Global Graph-Based Brain Functional Connectivity: Positive-Only Connectivity Weights. Modularity association with (a) age and (b) loneliness. Average shortest path length with (c) age and (d) loneliness

Supplementary Figure S3 Age and Graph-Based Brain Functional Connectivity Association Maps: Negative-Only Connectivity Weights. Color bar shows the z-score from Aspin-Welch's test for using exchangeability blocks to adjusted for four scanning sites and variance was estimated for each block, implemented in FSL PALM

Supplementary Figure S4 Association of Age and Global Graph-Based Brain Functional Connectivity: Negative-Only Connectivity Weights. Modularity association with (a) age and (b) loneliness. Average shortest path length with (c) age and (d) loneliness

Supplementary Table S1

Age and Nodal Strength Association Results

CAB-NP Label[a]	Glasser Label[b]	z[c]	-log10(p_{FWE})
Cingulo-Opercular-47_L-Ctx	Middle Insular Area	-4.55	3.7
Cingulo-Opercular-20_R-Ctx	Middle Insular Area	-4.5	3.7
Visual2-02_R-Ctx	Sixth Visual Area	-4.49	3.7
Auditory-07_R-Ctx	Auditory 4 Complex	-4.39	3.7
Default-33_R-Ctx	ParaHippocampal Area 2	-4.39	3.7
Auditory-15_L-Ctx	Auditory 4 Complex	-4.36	3.7
Cingulo-Opercular-21_R-Ctx	Frontal OPercular Area 1	-4.34	3.7
Cingulo-Opercular-25_R-Ctx	Area Posterior Insular 1	-4.29	3.7
Visual2-29_L-Ctx	Sixth Visual Area	-4.28	3.7
Cingulo-Opercular-22_R-Ctx	Frontal OPercular Area 3	-4.24	3.7
Auditory-04_R-Ctx	ParaBelt Complex	-4.23	3.7
Cingulo-Opercular-18_R-Ctx	Posterior Insular Area 2	-4.21	3.7
Visual2-01_R-Ctx	Medial Superior Temporal Area	-4.21	3.7
Auditory-12_L-Ctx	ParaBelt Complex	-4.16	3.7
Visual2-20_R-Ctx	VentroMedial Visual Area 1	-4.16	3.7
Cingulo-Opercular-38_L-Ctx	Anterior 24 prime	-4.14	3.7
Dorsal-Attention-19_L-Ctx	ParaHippocampal Area 3	-4.13	3.7
Visual2-53_L-Ctx	VentroMedial Visual Area 2	-4.13	3.7
Dorsal-Attention-07_R-Ctx	ParaHippocampal Area 3	-4.11	3.7
Language-06_R-Ctx	Auditory 5 Complex	-4.09	3.7
Cingulo-Opercular-44_L-Ctx	Area PFcm	-4.07	3.4
Visual1-06_L-Ctx	Dorsal Transitional Visual Area	-4.05	3.4
Cingulo-Opercular-39_L-Ctx	Area p32 prime	-4.05	3.4
Visual2-46_L-Ctx	Area V6A	-4.05	3.4
Visual1-03_R-Ctx	Dorsal Transitional Visual Area	-4.04	3.4
Cingulo-Opercular-01_R-Ctx	Frontal Eye Fields	-4.03	3.4
Visual2-47_L-Ctx	VentroMedial Visual Area 1	-4.02	3.4
Cingulo-Opercular-04_R-Ctx	Area 5m ventral	-4.02	3.4

Visual2-08_R-Ctx	Seventh Visual Area	-4.01	3.4
Cingulo-Opercular-52_L-Ctx	Area Posterior Insular 1	-4.01	3.4
Visual2-28_L-Ctx	Medial Superior Temporal Area	-4.01	3.4
Language-19_L-Ctx	Auditory 5 Complex	-3.99	3.4
Visual2-26_R-Ctx	VentroMedial Visual Area 2	-3.99	3.4
Default-25_R-Ctx	ParaHippocampal Area 1	-3.98	3.4
Language-07_R-Ctx	Area STSd posterior	-3.96	3.4
Cingulo-Opercular-16_R-Ctx	Area 43	-3.93	3.4
Default-64_L-Ctx	ParaHippocampal Area 1	-3.92	3.4
Default-71_L-Ctx	ParaHippocampal Area 2	-3.91	3.4
Cingulo-Opercular-17_R-Ctx	Area PFcm	-3.91	3.4
Auditory-03_R-Ctx	Area TA2	-3.91	3.4
Visual2-16_R-Ctx	Area Lateral IntraParietal ventral	-3.91	3.4
Visual2-27_R-Ctx	Ventral Visual Complex	-3.91	3.4
Cingulo-Opercular-49_L-Ctx	Frontal OPercular Area 3	-3.9	3.4
Visual2-19_R-Ctx	Area V6A	-3.89	3.22
Cingulo-Opercular-48_L-Ctx	Frontal OPercular Area 1	-3.87	3.1
Visual2-42_L-Ctx	Middle Temporal Area	-3.87	3.1
Cingulo-Opercular-43_L-Ctx	Area 43	-3.86	3.1
Visual2-38_L-Ctx	Area V3B	-3.84	3.1
Visual2-15_R-Cerebellum		-3.84	3.1
Visual2-22_R-Ctx	Area V4t	-3.82	3
Auditory-06_R-Ctx	Lateral Belt Complex	-3.82	3
Cingulo-Opercular-30_L-Ctx	Frontal Eye Fields	-3.81	3
Cingulo-Opercular-06_R-Ctx	Supplementary and Cingulate Eye Field	-3.8	3
Language-20_L-Ctx	Area STSd anterior	-3.79	3
Visual2-34_L-Ctx	Area V3A	-3.78	2.74
Visual2-54_L-Ctx	Ventral Visual Complex	-3.77	2.74
Posterior-Multimodal-02_R-Ctx	Superior Temporal Visual Area	-3.77	2.74
Somatomotor-08_R-Ctx	Area 7PC	-3.77	2.74
Somatomotor-13_R-Ctx	Area 6mp	-3.77	2.74

Default-63_L-Ctx	Hippocampus	-3.76	2.7
Visual2-43_L-Ctx	Area Lateral IntraParietal ventral	-3.76	2.59
Auditory-14_L-Ctx	Lateral Belt Complex	-3.76	2.59
Cingulo-Opercular-31_L-Ctx	Area 5m ventral	-3.75	2.59
Dorsal-Attention-04_R-Ctx	Area 6 anterior	-3.75	2.59
Visual2-15_R-Ctx	Middle Temporal Area	-3.75	2.59
Cingulo-Opercular-03_R-Ctx	PeriSylvian Language Area	-3.74	2.59
Somatomotor-15_R-Ctx	Area OP4/PV	-3.72	2.52
Visual2-25_R-Ctx	Area Lateral Occipital 3	-3.72	2.52
Cingulo-Opercular-27_R-Ctx	Para-Insular Area	-3.72	2.52
Default-18_R-Ctx	Area 9 anterior	-3.72	2.52
Language-13_L-Ctx	Superior Temporal Visual Area	-3.72	2.52
Visual2-35_L-Ctx	Seventh Visual Area	-3.71	2.47
Visual2-07_R-Ctx	Area V3A	-3.7	2.44
Cingulo-Opercular-32_L-Ctx	Area 23c	-3.7	2.44
Visual2-21_R-Ctx	VentroMedial Visual Area 3	-3.69	2.44
Visual2-09_R-Ctx	IntraParietal Sulcus Area 1	-3.68	2.44
Language-01_R-Ctx	Area 55b	-3.68	2.44
Somatomotor-18_R-Ctx	RetroInsular Cortex	-3.67	2.36
Language-08_R-Ctx	Area TemporoParietoOcci pital Junction 1	-3.67	2.32
Somatomotor-19_R-Ctx	Frontal OPercular Area 2	-3.67	2.3
Somatomotor-19_R-Hippocampus		-3.67	2.3
Visual2-48_L-Ctx	VentroMedial Visual Area 3	-3.66	2.3
Posterior-Multimodal-03_R-Ctx	Area TemporoParietoOcci pital Junction 2	-3.66	2.28
Language-16_L-Ctx	Area IFJa	-3.65	2.27
Orbito-Affective-01_R-Ctx	Pirform Cortex	-3.65	2.27
Visual2-30_L-Ctx	Second Visual Area	-3.64	2.27
Dorsal-Attention-09_L-Cerebellum		-3.64	2.27
Dorsal-Attention-20_L-Ctx	Area TE2 posterior	-3.64	2.27
Dorsal-Attention-14_L-Ctx	Medial IntraParietal Area	-3.64	2.27

Somatomotor-28_R-Thalamus		-3.64	2.27
Posterior-Multimodal-07_L-Ctx	Area TemporoParietoOcci pital Junction 3	-3.64	2.25
Visual2-36_L-Ctx	IntraParietal Sulcus Area 1	-3.63	2.24
Posterior-Multimodal-10_L-Cerebellum		-3.62	2.19
Cingulo-Opercular-11_R-Ctx	Area p32 prime	-3.61	2.15
Visual1-02_R-Ctx	ProStriate Area	-3.61	2.15
Visual2-08_L-Cerebellum		-3.6	2.13
Auditory-11_L-Ctx	Area TA2	-3.59	2.11
Default-15_R-Ctx	Area 9 Posterior	-3.59	2.11
Visual1-05_L-Ctx	ProStriate Area	-3.59	2.1
Cingulo-Opercular-45_L-Ctx	Posterior Insular Area 2	-3.59	2.09
Default-37_R-Ctx	Area STSv anterior	-3.59	2.09
Default-53_L-Ctx	Area 9 Posterior	-3.59	2.09
Visual2-06_R-Ctx	Eighth Visual Area	-3.58	2.05
Visual2-17_R-Ctx	Ventral IntraParietal Complex	-3.58	2.03
Auditory-10_L-Ctx	RetroInsular Cortex	-3.56	2.01
Somatomotor-36_L-Ctx	Area OP1/SII	-3.56	2
Somatomotor-28_L-Ctx	Area 7PC	-3.56	1.98
Visual2-03_R-Ctx	Second Visual Area	-3.56	1.98
Language-02_R-Ctx	Superior Frontal Language Area	-3.55	1.98
Cingulo-Opercular-10_R-Ctx	Anterior 24 prime	-3.55	1.97
Default-26_R-Ctx	Area STSd anterior	-3.55	1.97
Visual2-37_L-Ctx	Fusiform Face Complex	-3.54	1.96
Visual2-31_L-Ctx	Third Visual Area	-3.54	1.96
Dorsal-Attention-23_L-Ctx	Area IntraParietal 0	-3.54	1.96
Posterior-Multimodal-04_R-Ctx	Area TemporoParietoOcci pital Junction 3	-3.54	1.94
Cingulo-Opercular-07_R-Ctx	Area 6m anterior	-3.54	1.94
Language-17_L-Ctx	Area IFSp	-3.52	1.89
Visual2-10_R-Ctx	Fusiform Face Complex	-3.52	1.89
Default-14_R-Ctx	Area 8B Lateral	-3.51	1.87
Somatomotor-33_L-Ctx	Area 6mp	-3.5	1.84
Somatomotor-09_R-Ctx	Area 1	-3.5	1.82
Somatomotor-06_R-Ctx	Ventral Area 24d	-3.49	1.81
Somatomotor-26_L-Ctx	Ventral Area 24d	-3.49	1.81
Cingulo-Opercular-05_R-Ctx	Area 23c	-3.49	1.79

Language-10_L-Ctx	Area 55b	-3.48	1.77
Default-47_L-Ctx	Area 10r	-3.48	1.76
Visual2-49_L-Ctx	Area V4t	-3.47	1.75
Default-76_L-Ctx	Area STSv anterior	-3.46	1.73
Cingulo-Opercular-23_R-Ctx	Area PF opercular	-3.45	1.69
Somatomotor-12_R-Ctx	Dorsal area 6	-3.45	1.69
Language-21_L-Ctx	Area STSd posterior	-3.45	1.69
Visual2-52_L-Ctx	Area Lateral Occipital 3	-3.44	1.69
Somatomotor-10_R-Ctx	Area 2	-3.44	1.67
Language-04_R-Ctx	Area IFJa	-3.43	1.67
Somatomotor-23_L-Ctx	Area 5m	-3.43	1.63
Cingulo-Opercular-17_R-Cerebellum		-3.42	1.61

Note. [a]CAB-NP label per Cole-Anticevic Brain Network Parcellation v1.1.6.

[b]Glasser cortical label names were from Glasser, et al. (2016).

[c]*z*-score from Aspin-Welch's test for using exchangeability blocks to adjusted for four scanning sites and variance was estimated for each block, implemented in FSL PALM.

Supplementary Table S2

Age and Nodal Closeness Centrality Association Results

CAB-NP Label[a]	Glasser Label[b]	z[c]	-log10(p_{FWE})
Cingulo-Opercular-47_L-Ctx	Middle Insular Area	-4.82	3.7
Default-33_R-Ctx	ParaHippocampal Area 2	-4.67	3.7
Cingulo-Opercular-20_R-Ctx	Middle Insular Area	-4.62	3.7
Cingulo-Opercular-22_R-Ctx	Frontal OPercular Area 3	-4.49	3.7
Cingulo-Opercular-21_R-Ctx	Frontal OPercular Area 1	-4.45	3.7
Dorsal-Attention-07_R-Ctx	ParaHippocampal Area 3	-4.34	3.7
Visual2-53_L-Ctx	VentroMedial Visual Area 2	-4.33	3.7
Cingulo-Opercular-38_L-Ctx	Anterior 24 prime	-4.31	3.7
Dorsal-Attention-19_L-Ctx	ParaHippocampal Area 3	-4.31	3.7
Cingulo-Opercular-25_R-Ctx	Area Posterior Insular 1	-4.31	3.7
Cingulo-Opercular-49_L-Ctx	Frontal OPercular Area 3	-4.29	3.7
Cingulo-Opercular-18_R-Ctx	Posterior Insular Area 2	-4.28	3.7
Auditory-15_L-Ctx	Auditory 4 Complex	-4.22	3.7
Auditory-04_R-Ctx	ParaBelt Complex	-4.2	3.7
Visual2-20_R-Ctx	VentroMedial Visual Area 1	-4.2	3.7
Auditory-07_R-Ctx	Auditory 4 Complex	-4.2	3.7
Auditory-12_L-Ctx	ParaBelt Complex	-4.18	3.7
Visual2-26_R-Ctx	VentroMedial Visual Area 2	-4.17	3.7
Cingulo-Opercular-39_L-Ctx	Area p32 prime	-4.15	3.7
Language-07_R-Ctx	Area STSd posterior	-4.15	3.7
Default-71_L-Ctx	ParaHippocampal Area 2	-4.15	3.7
Visual2-02_R-Ctx	Sixth Visual Area	-4.14	3.7
Cingulo-Opercular-52_L-Ctx	Area Posterior Insular 1	-4.12	3.7
Language-19_L-Ctx	Auditory 5 Complex	-4.11	3.7
Cingulo-Opercular-48_L-Ctx	Frontal OPercular Area 1	-4.1	3.7
Cingulo-Opercular-27_R-Ctx	Para-Insular Area	-4.1	3.7
Visual2-47_L-Ctx	VentroMedial Visual Area 1	-4.05	3.7

Default-64_L-Ctx	ParaHippocampal Area 1	-4.02	3.7
Cingulo-Opercular-44_L-Ctx	Area PFcm	-4.02	3.7
Language-06_R-Ctx	Auditory 5 Complex	-4	3.7
Visual2-29_L-Ctx	Sixth Visual Area	-3.98	3.4
Somatomotor-19_R-Ctx	Frontal OPercular Area 2	-3.98	3.4
Visual2-01_R-Ctx	Medial Superior Temporal Area	-3.97	3.4
Default-53_L-Ctx	Area 9 Posterior	-3.97	3.4
Auditory-03_R-Ctx	Area TA2	-3.97	3.4
Cingulo-Opercular-04_R-Ctx	Area 5m ventral	-3.96	3.4
Cingulo-Opercular-16_R-Ctx	Area 43	-3.96	3.4
Default-25_R-Ctx	ParaHippocampal Area 1	-3.96	3.4
Posterior-Multimodal-02_R-Ctx	Superior Temporal Visual Area	-3.96	3.4
Language-02_R-Ctx	Superior Frontal Language Area	-3.96	3.4
Default-52_L-Ctx	Area 8B Lateral	-3.93	3.4
Auditory-06_R-Ctx	Lateral Belt Complex	-3.93	3.4
Default-15_R-Ctx	Area 9 Posterior	-3.93	3.4
Language-20_L-Ctx	Area STSd anterior	-3.93	3.4
Language-01_R-Ctx	Area 55b	-3.92	3.4
Cingulo-Opercular-06_R-Ctx	Supplementary and Cingulate Eye Field	-3.92	3.4
Cingulo-Opercular-17_R-Ctx	Area PFcm	-3.91	3.4
Default-63_L-Ctx	Hippocampus	-3.9	3.4
Posterior-Multimodal-10_L-Cerebellum		-3.89	3.4
Cingulo-Opercular-43_L-Ctx	Area 43	-3.89	3.4
Cingulo-Opercular-10_R-Ctx	Anterior 24 prime	-3.89	3.4
Default-37_R-Ctx	Area STSv anterior	-3.88	3.4
Visual1-06_L-Ctx	Dorsal Transitional Visual Area	-3.88	3.4
Auditory-14_L-Ctx	Lateral Belt Complex	-3.88	3.4
Default-14_R-Ctx	Area 8B Lateral	-3.88	3.4
Visual2-27_R-Ctx	Ventral Visual Complex	-3.88	3.4
Cingulo-Opercular-11_R-Ctx	Area p32 prime	-3.86	3.4
Somatomotor-19_R-Hippocampus		-3.86	3.4
Visual2-21_R-Ctx	VentroMedial Visual Area 3	-3.85	3.4

Default-26_R-Ctx	Area STSd anterior	-3.85	3.4
Cingulo-Opercular-19_R-Ctx	Frontal OPercular Area 4	-3.83	3.22
Visual1-03_R-Ctx	Dorsal Transitional Visual Area	-3.83	3.22
Visual2-08_L-Cerebellum		-3.82	3.22
Cingulo-Opercular-01_R-Ctx	Frontal Eye Fields	-3.82	3.22
Default-18_R-Ctx	Area 9 anterior	-3.82	3.22
Somatomotor-18_R-Ctx	RetroInsular Cortex	-3.81	3.22
Visual2-28_L-Ctx	Medial Superior Temporal Area	-3.81	3.1
Cingulo-Opercular-07_R-Ctx	Area 6m anterior	-3.8	3
Somatomotor-28_R-Thalamus		-3.8	3
Somatomotor-13_R-Ctx	Area 6mp	-3.79	3
Language-16_L-Ctx	Area IFJa	-3.79	3
Language-04_R-Ctx	Area IFJa	-3.79	3
Visual2-19_R-Ctx	Area V6A	-3.78	3
Language-13_L-Ctx	Superior Temporal Visual Area	-3.78	3
Cingulo-Opercular-03_R-Ctx	PeriSylvian Language Area	-3.78	3
Visual2-48_L-Ctx	VentroMedial Visual Area 3	-3.78	3
Cingulo-Opercular-45_L-Ctx	Posterior Insular Area 2	-3.78	3
Visual2-46_L-Ctx	Area V6A	-3.77	3
Cingulo-Opercular-30_L-Ctx	Frontal Eye Fields	-3.77	3
Visual2-42_L-Ctx	Middle Temporal Area	-3.76	3
Language-21_L-Ctx	Area STSd posterior	-3.76	3
Auditory-10_L-Ctx	RetroInsular Cortex	-3.75	3
Cingulo-Opercular-32_L-Ctx	Area 23c	-3.74	2.92
Language-08_R-Ctx	Area TemporoParietoOcci pital Junction 1	-3.74	2.92
Visual1-02_R-Ctx	ProStriate Area	-3.73	2.92
Somatomotor-38_L-Ctx	Frontal OPercular Area 2	-3.73	2.92
Cingulo-Opercular-31_L-Ctx	Area 5m ventral	-3.71	2.85
Visual2-54_L-Ctx	Ventral Visual Complex	-3.71	2.85
Visual2-15_R-Cerebellum		-3.71	2.85
Somatomotor-15_R-Ctx	Area OP4/PV	-3.71	2.85
Dorsal-Attention-04_R-Ctx	Area 6 anterior	-3.71	2.85
Visual1-05_L-Ctx	ProStriate Area	-3.7	2.85

Language-17_L-Ctx	Area IFSp	-3.69	2.85
Auditory-02_R-Ctx	Area 52	-3.68	2.8
Somatomotor-08_R-Ctx	Area 7PC	-3.68	2.8
Somatomotor-06_R-Ctx	Ventral Area 24d	-3.67	2.74
Dorsal-Attention-09_L-Cerebellum		-3.67	2.74
Dorsal-Attention-20_L-Ctx	Area TE2 posterior	-3.66	2.7
Somatomotor-14_R-Ctx	Ventral Area 6	-3.66	2.7
Language-10_L-Ctx	Area 55b	-3.66	2.7
Language-12_L-Ctx	Superior Frontal Language Area	-3.65	2.7
Visual2-22_R-Ctx	Area V4t	-3.65	2.7
Dorsal-Attention-16_R-Cerebellum		-3.63	2.7
Visual2-15_R-Ctx	Middle Temporal Area	-3.63	2.7
Default-23_L-Hippocampus		-3.63	2.7
Visual2-25_R-Ctx	Area Lateral Occipital 3	-3.62	2.7
Orbito-Affective-03_R-Ctx	posterior OFC Complex	-3.62	2.7
Visual2-08_R-Ctx	Seventh Visual Area	-3.61	2.7
Frontoparietal-04_R-Ctx	Area 33 prime	-3.61	2.7
Cingulo-Opercular-02_R-Ctx	Premotor Eye Field	-3.61	2.7
Posterior-Multimodal-05_L-Ctx	PreCuneus Visual Area	-3.61	2.7
Ventral-Multimodal-04_L-Ctx	Area TF	-3.61	2.7
Somatomotor-28_L-Ctx	Area 7PC	-3.6	2.7
Orbito-Affective-01_R-Ctx	Pirform Cortex	-3.6	2.7
Cingulo-Opercular-33_L-Ctx	Supplementary and Cingulate Eye Field	-3.59	2.7
Posterior-Multimodal-03_R-Ctx	Area TemporoParietoOcci pital Junction 2	-3.59	2.7
Cingulo-Opercular-37_L-Ctx	Area 33 prime	-3.59	2.7
Cingulo-Opercular-05_R-Ctx	Area 23c	-3.59	2.7
Visual2-38_L-Ctx	Area V3B	-3.59	2.7
Cingulo-Opercular-17_R-Cerebellum		-3.59	2.7
Auditory-11_L-Ctx	Area TA2	-3.58	2.62
Default-07_R-Ctx	Area a24	-3.58	2.62
Cingulo-Opercular-56_L-Ctx	Area posterior 24	-3.58	2.62
Visual2-16_R-Ctx	Area Lateral IntraParietal ventral	-3.58	2.62

Posterior-Multimodal-01_R-Ctx	PreCuneus Visual Area	-3.57	2.62
Default-76_L-Ctx	Area STSv anterior	-3.57	2.62
Posterior-Multimodal-07_L-Ctx	Area TemporoParietoOcci pital Junction 3	-3.57	2.62
Language-11_L-Ctx	PeriSylvian Language Area	-3.56	2.55
Visual2-43_L-Ctx	Area Lateral IntraParietal ventral	-3.56	2.55
Orbito-Affective-05_L-Ctx	Anterior Agranular Insula Complex	-3.55	2.55
Auditory-05_R-Ctx	Medial Belt Complex	-3.55	2.55
Somatomotor-26_L-Ctx	Ventral Area 24d	-3.55	2.52
Somatomotor-36_L-Ctx	Area OP1/SII	-3.54	2.52
Dorsal-Attention-17_L-Ctx	Area PFt	-3.54	2.49
Language-05_R-Ctx	Area STGa	-3.54	2.49
Cingulo-Opercular-09_R-Ctx	Area Posterior 24 prime	-3.53	2.49
Language-15_L-Ctx	Area 45	-3.53	2.49
Visual2-17_R-Ctx	Ventral IntraParietal Complex	-3.53	2.42
Posterior-Multimodal-04_R-Ctx	Area TemporoParietoOcci pital Junction 3	-3.52	2.42
Somatomotor-17_R-Ctx	Area OP2-3/VS	-3.51	2.38
Somatomotor-33_L-Ctx	Area 6mp	-3.51	2.34
Language-03_R-Ctx	Area 45	-3.51	2.34
Visual2-12_R-Ctx	Area Lateral Occipital 1	-3.51	2.3
Cingulo-Opercular-34_L-Ctx	Area 6m anterior	-3.5	2.3
Cingulo-Opercular-41_L-Ctx	Area 46	-3.5	2.3
Somatomotor-27_L-Thalamus		-3.5	2.28
Cingulo-Opercular-55_L-Ctx	Area anterior 32 prime	-3.5	2.28
Default-44_L-Ctx	Area a24	-3.5	2.28
Visual2-27_R-Hippocampus		-3.5	2.28
Dorsal-Attention-12_L-Ctx	Premotor Eye Field	-3.5	2.28
Somatomotor-03_R-Amygdala		-3.5	2.27
Somatomotor-07_R-Ctx	Lateral Area 7A	-3.49	2.25
Visual2-37_L-Ctx	Fusiform Face Complex	-3.49	2.25
Visual2-06_R-Ctx	Eighth Visual Area	-3.49	2.25
Auditory-13_L-Ctx	Medial Belt Complex	-3.49	2.25
Visual2-34_L-Ctx	Area V3A	-3.49	2.22

Visual2-09_R-Ctx	IntraParietal Sulcus Area 1	-3.48	2.22
Default-56_L-Ctx	Area 9 anterior	-3.48	2.22
Somatomotor-25_L-Ctx	Dorsal Area 24d	-3.48	2.21
Default-50_L-Ctx	Area 8Ad	-3.48	2.21
Default-55_L-Ctx	Area 47l (47 lateral)	-3.47	2.18
Dorsal-Attention-16_L-Ctx	Area 6 anterior	-3.47	2.18
Somatomotor-32_L-Ctx	Dorsal area 6	-3.47	2.17
Default-27_R-Ctx	Area STSv posterior	-3.47	2.17
Cingulo-Opercular-23_R-Ctx	Area PF opercular	-3.47	2.17
Visual2-35_L-Ctx	Seventh Visual Area	-3.46	2.17
Somatomotor-16_R-Ctx	Area OP1/SII	-3.46	2.17
Somatomotor-12_R-Ctx	Dorsal area 6	-3.46	2.17
Default-47_L-Ctx	Area 10r	-3.46	2.17
Ventral-Multimodal-02_R-Ctx	Area TF	-3.45	2.15
Somatomotor-34_L-Ctx	Ventral Area 6	-3.45	2.14
Dorsal-Attention-14_L-Ctx	Medial IntraParietal Area	-3.45	2.14
Somatomotor-35_L-Ctx	Area OP4/PV	-3.45	2.14
Cingulo-Opercular-08_R-Ctx	Medial Area 7A	-3.44	2.14
Frontoparietal-35_L-Ctx	Area IFJp	-3.44	2.13
Dorsal-Attention-03_R-Ctx	Area Lateral IntraParietal dorsal	-3.43	2.11
Language-07_R-Cerebellum		-3.43	2.11
Somatomotor-37_L-Ctx	Area OP2-3/VS	-3.42	2.11
Cingulo-Opercular-36_L-Ctx	Area Posterior 24 prime	-3.42	2.09
Frontoparietal-10_R-Ctx	Area IFJp	-3.42	2.09
Somatomotor-16_L-Hippocampus		-3.42	2.07
Auditory-14_L-Hippocampus		-3.41	2.06
Language-22_L-Ctx	Area TemporoParietoOcci pital Junction 1	-3.41	2.06
Frontoparietal-10_L-Caudate		-3.41	2.06
Visual2-49_L-Ctx	Area V4t	-3.41	2.06
Somatomotor-05_R-Ctx	Dorsal Area 24d	-3.41	2.06
Dorsal-Attention-05_R-Ctx	Area PFt	-3.41	2.03
Visual2-07_R-Ctx	Area V3A	-3.4	2.03
Visual2-10_R-Ctx	Fusiform Face Complex	-3.4	2
Somatomotor-20_R-Ctx	Insular Granular Complex	-3.39	1.99
Language-05_L-Cerebellum		-3.39	1.98
Somatomotor-24_L-Ctx	Area 5L	-3.39	1.98

Dorsal-Attention-22_L-Ctx	Area PGp	-3.39	1.97
Language-06_R-Cerebellum		-3.38	1.95
Somatomotor-10_R-Ctx	Area 2	-3.38	1.95
Visual2-36_L-Ctx	IntraParietal Sulcus Area 1	-3.38	1.95
Dorsal-Attention-02_R-Ctx	Medial IntraParietal Area	-3.37	1.93
Auditory-01_R-Ctx	Primary Auditory Cortex	-3.37	1.93
Posterior-Multimodal-18_R-Cerebellum		-3.37	1.93
Frontoparietal-11_R-Caudate		-3.37	1.93
Somatomotor-03_R-Ctx	Area 5m	-3.37	1.93
Default-24_R-Ctx	Hippocampus	-3.37	1.91
Dorsal-Attention-18_L-Ctx	Anterior IntraParietal Area	-3.36	1.91
Somatomotor-23_L-Ctx	Area 5m	-3.36	1.87
Somatomotor-09_R-Ctx	Area 1	-3.36	1.87
Visual2-13_R-Ctx	Area Lateral Occipital 2	-3.35	1.87
Default-12_R-Ctx	Area 8Ad	-3.35	1.86
Default-45_L-Ctx	Area dorsal 32	-3.35	1.85
Dorsal-Attention-23_L-Ctx	Area IntraParietal 0	-3.34	1.84
Visual2-51_L-Ctx	Area V3CD	-3.34	1.84
Cingulo-Opercular-26_R-Ctx	Area Frontal Opercular 5	-3.34	1.84
Default-51_L-Ctx	Area 9 Middle	-3.33	1.84
Default-65_L-Ctx	Area STSv posterior	-3.33	1.81
Somatomotor-27_L-Ctx	Lateral Area 7A	-3.33	1.8
Visual2-44_L-Ctx	Ventral IntraParietal Complex	-3.33	1.8
Cingulo-Opercular-46_L-Ctx	Frontal OPercular Area 4	-3.32	1.79
Visual2-39_L-Ctx	Area Lateral Occipital 1	-3.31	1.77
Dorsal-Attention-13_L-Ctx	Lateral Area 7P	-3.31	1.77
Posterior-Multimodal-11_L-Cerebellum		-3.31	1.77
Frontoparietal-36_L-Ctx	Area IFSa	-3.3	1.75
Visual2-52_L-Ctx	Area Lateral Occipital 3	-3.3	1.75
Somatomotor-30_L-Ctx	Area 2	-3.3	1.74
Visual2-23_R-Ctx	Area FST	-3.3	1.74
Visual2-30_L-Ctx	Second Visual Area	-3.29	1.73
Visual2-03_R-Ctx	Second Visual Area	-3.29	1.73
Visual2-33_L-Ctx	Eighth Visual Area	-3.28	1.73
Dorsal-Attention-01_R-Ctx	Lateral Area 7P	-3.28	1.72
Cingulo-Opercular-35_L-Ctx	Medial Area 7A	-3.28	1.72

Visual2-31_L-Ctx	Third Visual Area	-3.27	1.69
Somatomotor-01_R-Ctx	Primary Motor Cortex	-3.27	1.69
Cingulo-Opercular-28_R-Ctx	Area anterior 32 prime	-3.27	1.69
Somatomotor-04_R-Ctx	Area 5L	-3.26	1.67
Somatomotor-02_R-Ctx	Primary Sensory Cortex	-3.26	1.66
Visual2-10_L-Cerebellum		-3.25	1.65
Dorsal-Attention-10_R-Ctx	Area PGp	-3.25	1.64
Somatomotor-21_L-Ctx	Primary Motor Cortex	-3.25	1.64
Dorsal-Attention-11_R-Ctx	Area IntraParietal 0	-3.24	1.63
Dorsal-Attention-06_R-Ctx	Anterior IntraParietal Area	-3.24	1.61
Visual2-11_R-Ctx	Area V3B	-3.24	1.61

Note. [a]CAB-NP label per Cole-Anticevic Brain Network Parcellation v1.1.6.

[b]Glasser cortical label names were from Glasser, et al. (2016).

[c]*z*-score from Aspin-Welch's test for using exchangeability blocks to adjusted for four scanning sites and variance was estimated for each block, implemented in FSL PALM.

Supplementary Table S3

Age and Nodal Betweenness Centrality Association Results

CAB-NP Label[a]	Glasser Label[b]	z[c]	-log10(p_{FWE})
Visual1-51_L-Putamen		4.45	1.91
Somatomotor-25_L-Putamen		4.39	1.8

Note. [a]CAB-NP label per Cole-Anticevic Brain Network Parcellation v1.1.6.

[b]Glasser cortical label names were from Glasser, et al. (2016).

[c]*z*-score from Aspin-Welch's test for using exchangeability blocks to adjusted for four scanning sites and variance was estimated for each block, implemented in FSL PALM.

Supplementary Table S4

Age and Nodal Eigenvector Centrality Association Results

CAB-NP Label[a]	Glasser Label[b]	z[c]	-log10(p_{FWE})
	Positive Association		
Frontoparietal-27_R-Cerebellum		4.57	3.7
Frontoparietal-24_R-Ctx	Area PFm Complex	4.55	3.7
Frontoparietal-48_L-Ctx	Area PFm Complex	4.39	3.7
Frontoparietal-15_R-Ctx	Area 11l	4.24	3.7
Frontoparietal-40_L-Ctx	Area 11l	4.2	3.7
Frontoparietal-13_L-Cerebellum		4.13	3.7
Somatomotor-22_L-Pallidum		4.11	3.7
Frontoparietal-23_R-Ctx	Area IntraParietal 1	4.1	3.7
Cingulo-Opercular-25_L-Diencephalon		4.09	3.7
Frontoparietal-37_R-Cerebellum		4.07	3.7
Frontoparietal-07_R-Ctx	Area 8C	4.04	3.7
Frontoparietal-09_R-Ctx	Area anterior 47r	3.98	3.7
Auditory-06_L-Cerebellum		3.95	3.7
Frontoparietal-39_L-Diencephalon		3.95	3.7
Frontoparietal-46_L-Ctx	Area IntraParietal 2	3.95	3.7
Default-03_R-Ctx	Area 23d	3.95	3.7
Frontoparietal-30_L-Ctx	Parieto-Occipital Sulcus Area 2	3.89	3.7
Visual1-47_L-Pallidum		3.87	3.7
Default-40_L-Ctx	Area 23d	3.84	3.4
Visual1-11_L-Brainstem		3.84	3.4
Visual1-48_R-Pallidum		3.83	3.22
Frontoparietal-33_L-Ctx	Area 8C	3.83	3.22
Frontoparietal-04_R-Brainstem		3.83	3.22
Default-21_R-Diencephalon		3.82	3.22
Visual1-28_L-Cerebellum		3.82	3.1
Auditory-31_R-Thalamus		3.82	3
Auditory-20_L-Pallidum		3.8	3
Frontoparietal-22_R-Ctx	Area IntraParietal 2	3.78	2.85
Ventral-Multimodal-04_R-Hippocampus		3.76	2.8
Posterior-Multimodal-05_L-Brainstem		3.75	2.74
Default-17_R-Cerebellum		3.74	2.66
Default-32_R-Ctx	Area PGs	3.73	2.62

Dorsal-Attention-01_L-Amygdala		3.7	2.62
Frontoparietal-12_R-Ctx	Area posterior 9-46v	3.66	2.44
Somatomotor-15_L-Diencephalon		3.61	2.34
Frontoparietal-47_L-Ctx	Area IntraParietal 1	3.59	2.3
Auditory-08_L-Diencephalon		3.59	2.3
Visual1-49_R-Pallidum		3.58	2.27
Frontoparietal-50_L-Ctx	Area posterior 47r	3.58	2.24
Frontoparietal-19_L-Cerebellum		3.58	2.24
Auditory-11_R-Diencephalon		3.55	2.15
Orbito-Affective-12_R-Pallidum		3.55	2.15
Frontoparietal-36_R-Cerebellum		3.53	2.08
Visual1-02_L-Accumbens		3.52	2.03
Frontoparietal-47_L-Thalamus		3.52	2.02
Frontoparietal-45_L-Ctx	Area TE1 posterior	3.5	1.93
Orbito-Affective-03_L-Caudate		3.49	1.89
Frontoparietal-34_L-Ctx	Area anterior 47r	3.48	1.82
Default-16_R-Cerebellum		3.47	1.8
Auditory-07_R-Cerebellum		3.45	1.75
Cingulo-Opercular-06_L-Brainstem		3.44	1.7
Frontoparietal-42_L-Pallidum		3.44	1.7
Cingulo-Opercular-26_L-Diencephalon		3.42	1.66
	Negative Association		
Visual2-20_R-Ctx	VentroMedial Visual Area 1	-4.8	3.7
Visual2-02_R-Ctx	Sixth Visual Area	-4.64	3.7
Auditory-07_R-Ctx	Auditory 4 Complex	-4.55	3.7
Auditory-15_L-Ctx	Auditory 4 Complex	-4.38	3.7
Auditory-04_R-Ctx	ParaBelt Complex	-4.31	3.7
Visual2-26_R-Ctx	VentroMedial Visual Area 2	-4.3	3.7
Auditory-12_L-Ctx	ParaBelt Complex	-4.3	3.7
Visual2-29_L-Ctx	Sixth Visual Area	-4.16	3.7
Visual2-47_L-Ctx	VentroMedial Visual Area 1	-4.14	3.7
Cingulo-Opercular-18_R-Ctx	Posterior Insular Area 2	-4.03	3.7
Visual2-53_L-Ctx	VentroMedial Visual Area 2	-3.97	3.7
Auditory-03_R-Ctx	Area TA2	-3.83	3.22

Auditory-06_R-Ctx	Lateral Belt Complex	-3.82	3
Language-06_R-Ctx	Auditory 5 Complex	-3.79	2.92
Cingulo-Opercular-04_R-Ctx	Area 5m ventral	-3.76	2.74
Auditory-14_L-Ctx	Lateral Belt Complex	-3.73	2.62
Cingulo-Opercular-44_L-Ctx	Area PFcm	-3.73	2.62
Visual2-01_R-Ctx	Medial Superior Temporal Area	-3.72	2.62
Somatomotor-18_R-Ctx	RetroInsular Cortex	-3.64	2.42
Auditory-10_L-Ctx	RetroInsular Cortex	-3.55	2.15
Visual1-06_L-Ctx	Dorsal Transitional Visual Area	-3.54	2.12
Visual1-03_R-Ctx	Dorsal Transitional Visual Area	-3.52	2.03
Visual1-02_R-Ctx	ProStriate Area	-3.5	1.94
Cingulo-Opercular-21_R-Ctx	Frontal OPercular Area 1	-3.5	1.92
Language-19_L-Ctx	Auditory 5 Complex	-3.49	1.86
Dorsal-Attention-07_R-Ctx	ParaHippocampal Area 3	-3.48	1.84
Default-33_R-Ctx	ParaHippocampal Area 2	-3.48	1.81
Visual2-27_R-Ctx	Ventral Visual Complex	-3.46	1.79
Visual1-05_L-Ctx	ProStriate Area	-3.44	1.7

Note. [a]CAB-NP label per Cole-Anticevic Brain Network Parcellation v1.1.6.

[b]Glasser cortical label names were from Glasser, et al. (2016).

[c]*z*-score from Aspin-Welch's test for using exchangeability blocks to adjusted for four scanning sites and variance was estimated for each block, implemented in FSL PALM.

Supplementary Table S5

Age and Nodal Clustering Coefficient Association Results

CAB-NP Label[a]	Glasser Label[b]	z[c]	-log10(p_{FWE})
Visual2-02_R-Ctx	Sixth Visual Area	-4.61	3.7
Auditory-15_L-Ctx	Auditory 4 Complex	-4.45	3.7
Visual2-29_L-Ctx	Sixth Visual Area	-4.42	3.7
Cingulo-Opercular-47_L-Ctx	Middle Insular Area	-4.42	3.7
Visual2-01_R-Ctx	Medial Superior Temporal Area	-4.4	3.7
Auditory-07_R-Ctx	Auditory 4 Complex	-4.39	3.7
Cingulo-Opercular-20_R-Ctx	Middle Insular Area	-4.37	3.7
Visual2-20_R-Ctx	VentroMedial Visual Area 1	-4.34	3.7
Default-33_R-Ctx	ParaHippocampal Area 2	-4.31	3.7
Cingulo-Opercular-18_R-Ctx	Posterior Insular Area 2	-4.31	3.7
Auditory-04_R-Ctx	ParaBelt Complex	-4.29	3.7
Cingulo-Opercular-38_L-Ctx	Anterior 24 prime	-4.27	3.7
Cingulo-Opercular-21_R-Ctx	Frontal OPercular Area 1	-4.27	3.7
Auditory-12_L-Ctx	ParaBelt Complex	-4.27	3.7
Visual2-53_L-Ctx	VentroMedial Visual Area 2	-4.24	3.7
Cingulo-Opercular-25_R-Ctx	Area Posterior Insular 1	-4.23	3.7
Cingulo-Opercular-22_R-Ctx	Frontal OPercular Area 3	-4.23	3.7
Visual2-28_L-Ctx	Medial Superior Temporal Area	-4.21	3.7
Language-19_L-Ctx	Auditory 5 Complex	-4.21	3.7
Visual2-46_L-Ctx	Area V6A	-4.2	3.7
Visual2-47_L-Ctx	VentroMedial Visual Area 1	-4.19	3.7
Language-06_R-Ctx	Auditory 5 Complex	-4.17	3.7
Cingulo-Opercular-04_R-Ctx	Area 5m ventral	-4.14	3.7
Visual2-42_L-Ctx	Middle Temporal Area	-4.14	3.7
Default-25_R-Ctx	ParaHippocampal Area 1	-4.13	3.7
Dorsal-Attention-07_R-Ctx	ParaHippocampal Area 3	-4.13	3.7
Dorsal-Attention-19_L-Ctx	ParaHippocampal Area 3	-4.1	3.7
Auditory-03_R-Ctx	Area TA2	-4.1	3.7

Visual2-22_R-Ctx	Area V4t	-4.1	3.7
Visual2-26_R-Ctx	VentroMedial Visual Area 2	-4.09	3.7
Visual2-08_R-Ctx	Seventh Visual Area	-4.08	3.4
Cingulo-Opercular-39_L-Ctx	Area p32 prime	-4.06	3.4
Visual2-15_R-Ctx	Middle Temporal Area	-4.06	3.4
Cingulo-Opercular-44_L-Ctx	Area PFcm	-4.04	3.4
Visual2-19_R-Ctx	Area V6A	-4.04	3.4
Visual1-06_L-Ctx	Dorsal Transitional Visual Area	-4.02	3.22
Cingulo-Opercular-52_L-Ctx	Area Posterior Insular 1	-4.01	3.22
Default-64_L-Ctx	ParaHippocampal Area 1	-4.01	3.22
Somatomotor-13_R-Ctx	Area 6mp	-4.01	3.22
Visual1-03_R-Ctx	Dorsal Transitional Visual Area	-4	3.22
Somatomotor-08_R-Ctx	Area 7PC	-3.99	3.22
Visual2-34_L-Ctx	Area V3A	-3.96	3.22
Cingulo-Opercular-27_R-Ctx	Para-Insular Area	-3.96	3.22
Language-20_L-Ctx	Area STSd anterior	-3.95	3.22
Visual2-27_R-Ctx	Ventral Visual Complex	-3.94	3.22
Visual2-16_R-Ctx	Area Lateral IntraParietal ventral	-3.92	3.22
Cingulo-Opercular-01_R-Ctx	Frontal Eye Fields	-3.92	3.22
Auditory-14_L-Ctx	Lateral Belt Complex	-3.92	3.22
Auditory-06_R-Ctx	Lateral Belt Complex	-3.92	3.1
Visual2-54_L-Ctx	Ventral Visual Complex	-3.9	3.1
Cingulo-Opercular-48_L-Ctx	Frontal OPercular Area 1	-3.9	3.1
Cingulo-Opercular-16_R-Ctx	Area 43	-3.9	3
Cingulo-Opercular-17_R-Ctx	Area PFcm	-3.89	3
Orbito-Affective-01_R-Ctx	Pirform Cortex	-3.89	3
Visual1-02_R-Ctx	ProStriate Area	-3.89	3
Language-13_L-Ctx	Superior Temporal Visual Area	-3.89	3
Visual2-38_L-Ctx	Area V3B	-3.89	3
Default-71_L-Ctx	ParaHippocampal Area 2	-3.88	3
Cingulo-Opercular-49_L-Ctx	Frontal OPercular Area 3	-3.88	3
Language-07_R-Ctx	Area STSd posterior	-3.87	3

Somatomotor-19_R-Ctx	Frontal OPercular Area 2	-3.85	3
Visual2-15_R-Cerebellum		-3.84	3
Cingulo-Opercular-06_R-Ctx	Supplementary and Cingulate Eye Field	-3.84	3
Posterior-Multimodal-02_R-Ctx	Superior Temporal Visual Area	-3.83	2.92
Visual2-25_R-Ctx	Area Lateral Occipital 3	-3.83	2.92
Somatomotor-28_L-Ctx	Area 7PC	-3.83	2.92
Cingulo-Opercular-43_L-Ctx	Area 43	-3.83	2.92
Visual2-35_L-Ctx	Seventh Visual Area	-3.83	2.92
Dorsal-Attention-04_R-Ctx	Area 6 anterior	-3.83	2.92
Visual2-43_L-Ctx	Area Lateral IntraParietal ventral	-3.83	2.92
Somatomotor-28_R-Thalamus		-3.81	2.92
Default-63_L-Ctx	Hippocampus	-3.81	2.92
Somatomotor-12_R-Ctx	Dorsal area 6	-3.81	2.92
Visual1-05_L-Ctx	ProStriate Area	-3.81	2.92
Visual2-17_R-Ctx	Ventral IntraParietal Complex	-3.81	2.92
Visual2-48_L-Ctx	VentroMedial Visual Area 3	-3.8	2.92
Cingulo-Opercular-30_L-Ctx	Frontal Eye Fields	-3.79	2.92
Cingulo-Opercular-31_L-Ctx	Area 5m ventral	-3.79	2.92
Posterior-Multimodal-03_R-Ctx	Area TemporoParietoOcci pital Junction 2	-3.79	2.92
Visual2-07_R-Ctx	Area V3A	-3.79	2.92
Default-18_R-Ctx	Area 9 anterior	-3.77	2.92
Auditory-11_L-Ctx	Area TA2	-3.77	2.92
Somatomotor-09_R-Ctx	Area 1	-3.77	2.92
Somatomotor-15_R-Ctx	Area OP4/PV	-3.77	2.92
Somatomotor-33_L-Ctx	Area 6mp	-3.76	2.92
Visual2-49_L-Ctx	Area V4t	-3.76	2.85
Cingulo-Opercular-45_L-Ctx	Posterior Insular Area 2	-3.75	2.8
Somatomotor-05_R-Ctx	Dorsal Area 24d	-3.75	2.8
Somatomotor-19_R-Hippocampus		-3.74	2.74
Posterior-Multimodal-07_L-Ctx	Area TemporoParietoOcci pital Junction 3	-3.74	2.74
Visual2-21_R-Ctx	VentroMedial Visual Area 3	-3.74	2.74

Cingulo-Opercular-10_R-Ctx	Anterior 24 prime	-3.73	2.74
Visual2-09_R-Ctx	IntraParietal Sulcus Area 1	-3.73	2.7
Visual2-36_L-Ctx	IntraParietal Sulcus Area 1	-3.73	2.7
Somatomotor-23_L-Ctx	Area 5m	-3.72	2.7
Somatomotor-36_L-Ctx	Area OP1/SII	-3.72	2.7
Visual2-08_L-Cerebellum		-3.72	2.7
Somatomotor-18_R-Ctx	RetroInsular Cortex	-3.72	2.7
Somatomotor-06_R-Ctx	Ventral Area 24d	-3.72	2.7
Somatomotor-32_L-Ctx	Dorsal area 6	-3.71	2.7
Posterior-Multimodal-04_R-Ctx	Area TemporoParietoOcci pital Junction 3	-3.71	2.7
Dorsal-Attention-09_L-Cerebellum		-3.69	2.7
Visual2-52_L-Ctx	Area Lateral Occipital 3	-3.69	2.7
Somatomotor-26_L-Ctx	Ventral Area 24d	-3.69	2.7
Somatomotor-24_L-Ctx	Area 5L	-3.68	2.66
Visual2-30_L-Ctx	Second Visual Area	-3.68	2.66
Somatomotor-07_R-Ctx	Lateral Area 7A	-3.67	2.62
Cingulo-Opercular-11_R-Ctx	Area p32 prime	-3.67	2.62
Somatomotor-03_R-Ctx	Area 5m	-3.65	2.62
Visual2-37_L-Ctx	Fusiform Face Complex	-3.65	2.62
Auditory-10_L-Ctx	RetroInsular Cortex	-3.64	2.59
Cingulo-Opercular-03_R-Ctx	PeriSylvian Language Area	-3.63	2.59
Visual2-44_L-Ctx	Ventral IntraParietal Complex	-3.63	2.59
Dorsal-Attention-14_L-Ctx	Medial IntraParietal Area	-3.63	2.59
Cingulo-Opercular-32_L-Ctx	Area 23c	-3.63	2.55
Somatomotor-02_R-Ctx	Primary Sensory Cortex	-3.62	2.55
Default-37_R-Ctx	Area STSv anterior	-3.62	2.52
Cingulo-Opercular-17_R-Cerebellum		-3.62	2.52
Language-16_L-Ctx	Area IFJa	-3.61	2.52
Default-26_R-Ctx	Area STSd anterior	-3.61	2.47
Language-01_R-Ctx	Area 55b	-3.61	2.44
Visual2-06_R-Ctx	Eighth Visual Area	-3.6	2.44
Cingulo-Opercular-23_R-Ctx	Area PF opercular	-3.6	2.44
Somatomotor-16_R-Ctx	Area OP1/SII	-3.59	2.44
Somatomotor-10_R-Ctx	Area 2	-3.59	2.44
Somatomotor-29_L-Ctx	Area 1	-3.59	2.44

Dorsal-Attention-20_L-Ctx	Area TE2 posterior	-3.59	2.42
Somatomotor-25_L-Ctx	Dorsal Area 24d	-3.59	2.42
Somatomotor-04_R-Ctx	Area 5L	-3.58	2.42
Language-08_R-Ctx	Area TemporoParietoOcci pital Junction 1	-3.57	2.38
Visual2-03_R-Ctx	Second Visual Area	-3.57	2.36
Somatomotor-27_L-Ctx	Lateral Area 7A	-3.57	2.36
Dorsal-Attention-16_L-Ctx	Area 6 anterior	-3.57	2.36
Visual2-10_R-Ctx	Fusiform Face Complex	-3.57	2.36
Visual2-12_R-Ctx	Area Lateral Occipital 1	-3.57	2.34
Language-05_R-Ctx	Area STGa	-3.56	2.34
Posterior-Multimodal-10_L-Cerebellum		-3.56	2.34
Default-76_L-Ctx	Area STSv anterior	-3.55	2.34
Visual2-31_L-Ctx	Third Visual Area	-3.55	2.32
Dorsal-Attention-23_L-Ctx	Area IntraParietal 0	-3.55	2.32
Somatomotor-30_L-Ctx	Area 2	-3.55	2.32
Somatomotor-14_R-Ctx	Ventral Area 6	-3.54	2.32
Visual2-23_R-Ctx	Area FST	-3.54	2.3
Language-02_R-Ctx	Superior Frontal Language Area	-3.53	2.28
Somatomotor-17_R-Ctx	Area OP2-3/VS	-3.53	2.27
Somatomotor-22_L-Ctx	Primary Sensory Cortex	-3.52	2.27
Auditory-02_R-Ctx	Area 52	-3.52	2.27
Language-21_L-Ctx	Area STSd posterior	-3.52	2.27
Somatomotor-01_R-Ctx	Primary Motor Cortex	-3.52	2.27
Dorsal-Attention-12_L-Ctx	Premotor Eye Field	-3.52	2.27
Cingulo-Opercular-09_R-Ctx	Area Posterior 24 prime	-3.51	2.25
Somatomotor-21_L-Ctx	Primary Motor Cortex	-3.5	2.21
Somatomotor-27_L-Thalamus		-3.5	2.21
Visual2-39_L-Ctx	Area Lateral Occipital 1	-3.5	2.18
Dorsal-Attention-02_R-Ctx	Medial IntraParietal Area	-3.5	2.15
Default-15_R-Ctx	Area 9 Posterior	-3.49	2.15
Visual2-13_R-Ctx	Area Lateral Occipital 2	-3.49	2.15
Default-47_L-Ctx	Area 10r	-3.49	2.15
Visual2-51_L-Ctx	Area V3CD	-3.49	2.13
Language-17_L-Ctx	Area IFSp	-3.49	2.13

Ventral-Multimodal-04_L-Ctx	Area TF	-3.48	2.1
Default-24_R-Ctx	Hippocampus	-3.48	2.1
Cingulo-Opercular-07_R-Ctx	Area 6m anterior	-3.48	2.09
Cingulo-Opercular-37_L-Ctx	Area 33 prime	-3.48	2.08
Visual2-50_L-Ctx	Area FST	-3.47	2.05
Somatomotor-38_L-Ctx	Frontal OPercular Area 2	-3.46	2.02
Language-10_L-Ctx	Area 55b	-3.46	2.02
Visual2-11_R-Ctx	Area V3B	-3.45	1.97
Default-23_L-Hippocampus		-3.45	1.95
Posterior-Multimodal-01_R-Ctx	PreCuneus Visual Area	-3.44	1.93
Dorsal-Attention-03_R-Ctx	Area Lateral IntraParietal dorsal	-3.43	1.92
Visual2-33_L-Ctx	Eighth Visual Area	-3.42	1.9
Orbito-Affective-04_L-Ctx	Pirform Cortex	-3.41	1.89
Cingulo-Opercular-33_L-Ctx	Supplementary and Cingulate Eye Field	-3.41	1.89
Cingulo-Opercular-36_L-Ctx	Area Posterior 24 prime	-3.4	1.88
Posterior-Multimodal-05_L-Ctx	PreCuneus Visual Area	-3.4	1.87
Dorsal-Attention-11_R-Ctx	Area IntraParietal 0	-3.4	1.86
Dorsal-Attention-17_L-Ctx	Area PFt	-3.4	1.84
Cingulo-Opercular-08_R-Ctx	Medial Area 7A	-3.39	1.84
Cingulo-Opercular-02_R-Ctx	Premotor Eye Field	-3.38	1.8
Somatomotor-35_L-Ctx	Area OP4/PV	-3.38	1.8
Language-04_R-Ctx	Area IFJa	-3.38	1.8
Cingulo-Opercular-05_R-Ctx	Area 23c	-3.38	1.8
Somatomotor-31_L-Ctx	Area 3a	-3.38	1.8
Language-22_L-Ctx	Area TemporoParietoOcci pital Junction 1	-3.38	1.79
Somatomotor-37_L-Ctx	Area OP2-3/VS	-3.37	1.75
Dorsal-Attention-16_R-Cerebellum		-3.35	1.72
Visual2-05_R-Ctx	Fourth Visual Area	-3.35	1.72
Frontoparietal-04_R-Ctx	Area 33 prime	-3.35	1.69
Visual2-27_R-Hippocampus		-3.34	1.69
Dorsal-Attention-18_L-Ctx	Anterior IntraParietal Area	-3.34	1.69
Dorsal-Attention-22_L-Ctx	Area PGp	-3.34	1.69
Visual2-10_L-Cerebellum		-3.34	1.69

Somatomotor-03_R-Amygdala		-3.34	1.69
Default-53_L-Ctx	Area 9 Posterior	-3.34	1.67
Somatomotor-20_R-Ctx	Insular Granular Complex	-3.33	1.67
Visual2-04_R-Ctx	Third Visual Area	-3.32	1.67
Cingulo-Opercular-56_L-Ctx	Area posterior 24	-3.31	1.63
Ventral-Multimodal-02_R-Ctx	Area TF	-3.31	1.62
Default-62_L-Ctx	PreSubiculum	-3.31	1.62
Default-14_R-Ctx	Area 8B Lateral	-3.31	1.62
Dorsal-Attention-13_L-Ctx	Lateral Area 7P	-3.3	1.61
Dorsal-Attention-05_R-Ctx	Area PFt	-3.3	1.61
Visual2-32_L-Ctx	Fourth Visual Area	-3.29	1.6

Note. [a]CAB-NP label per Cole-Anticevic Brain Network Parcellation v1.1.6.

[b]Glasser cortical label names were from Glasser, et al. (2016).

[c]*z*-score from Aspin-Welch's test for using exchangeability blocks to adjusted for four scanning sites and variance was estimated for each block, implemented in FSL PALM.

Supplementary Table S6

Age and Nodal Participation Coefficient Association Results

CAB-NP Label[a]	Glasser Label[b]	z[c]	-log10(p_{FWE})
Positive Association			
Cingulo-Opercular-47_L-Ctx	Middle Insular Area	3.93	3.22
Cingulo-Opercular-20_R-Ctx	Middle Insular Area	3.93	3.22
Cingulo-Opercular-49_L-Ctx	Frontal OPercular Area 3	3.88	3.1
Cingulo-Opercular-11_R-Ctx	Area p32 prime	3.83	2.85
Somatomotor-18_L-Hippocampus		3.77	2.85
Cingulo-Opercular-22_R-Ctx	Frontal OPercular Area 3	3.69	2.47
Somatomotor-28_R-Thalamus		3.65	2.34
Cingulo-Opercular-46_L-Ctx	Frontal OPercular Area 4	3.65	2.32
Cingulo-Opercular-19_R-Ctx	Frontal OPercular Area 4	3.65	2.32
Somatomotor-16_L-Hippocampus		3.64	2.3
Somatomotor-27_L-Thalamus		3.6	2.19
Cingulo-Opercular-07_R-Ctx	Area 6m anterior	3.52	1.84
Visual2-20_R-Ctx	VentroMedial Visual Area 1	3.47	1.62
Auditory-09_L-Ctx	Area 52	3.46	1.61
Negative Association			
Visual1-56_L-Thalamus		-3.78	2.85
Visual1-02_R-Ctx	ProStriate Area	-3.62	2.24

Note. [a]CAB-NP label per Cole-Anticevic Brain Network Parcellation v1.1.6.

[b]Glasser cortical label names were from Glasser, et al. (2016).

[c]*z*-score from Aspin-Welch's test for using exchangeability blocks to adjusted for four scanning sites and variance was estimated for each block, implemented in FSL PALM.

Supplementary Table S7

Summary of Age and Graph-Based Brain Functional Connectivity Results: Positive-Only Connectivity Weights

Graph Measure	Positive Association			Negative Association		
	Cortical	Subcortical	CAB-NP Networks	Cortical	Subcortical	CAB-NP Networks
Normalized Strength	--	--	--	125	5	AUD, CON, DAN, DMN, LAN, ORA, PMM, SMN, VIS1, VIS2
Closeness Centrality	--	--	--	231	26	Across all 12 networks
Betweenness Centrality	1	2	DMN, SMN, VIS2	--	--	--
Eigenvector Centrality	23	39	AUD, CON, DAN, DMN, FPN, ORA, SMN, VIS1	29	1	AUD, CON, DMN, LAN, SMN, VIS1, VIS2
Clustering Coefficient	--	--	--	197	17	AUD, CON, DAN, DMN, LAN, ORA, PMM, SMN, VIS1, VIS2, VMN
Participation Coefficient	1	4	CON, SMN	--	--	--

Note. 12 CAB-NP Networks: AUD: Auditory Network; CON: Cingulo-Opercular Network; DAN: Dorsal Attention Network; DMN: Default Mode Network; FPN: Frontoparietal Network; LAN: Language Network; ORA: Orbito-Affective Network; PMM: Posterior Multimodal Network; SMN: Somatomotor Network; VIS1: Primary Visual Network; VIS2: Secondary Visual Network; VMM: Ventral Multimodal Network [3].

Supplementary Table S8

Summary of Age and Graph-Based Brain Functional Connectivity Results: Negative-Only Connectivity Weights

Graph Measure	Positive Association			Negative Association		
	Cortical	Subcortical	CAB-NP Networks	Cortical	Subcortical	CAB-NP Networks
Normalized Strength	--	--	--	--	2	DMN, FPN
Closeness Centrality	--	--	--	10	--	CON, DAN, DMN, LAN, ORA, VIS2
Betweenness Centrality	--	--	--	--	--	--
Eigenvector Centrality	--	1	VIS1	--	1	FPN
Clustering Coefficient	--	--	--	--	--	--
Participation Coefficient	--	--	--	1	1	SMN, VIS2

Note. 12 CAB-NP Networks: AUD: Auditory Network; CON: Cingulo-Opercular Network; DAN: Dorsal Attention Network; DMN: Default Mode Network; FPN: Frontoparietal Network; LAN: Language Network; ORA: Orbito-Affective Network; PMM: Posterior Multimodal Network; SMN: Somatomotor Network; VIS1: Primary Visual Network; VIS2: Secondary Visual Network; VMM: Ventral Multimodal Network [3].

Supplementary Figure S1

Age and Graph-Based Brain Functional Connectivity Association Maps: Positive-Only Connectivity Weights. Color bar shows the z-score from Aspin-Welch's test for using exchangeability blocks to adjusted for four scanning sites and variance was estimated for each block, implemented in FSL PALM.

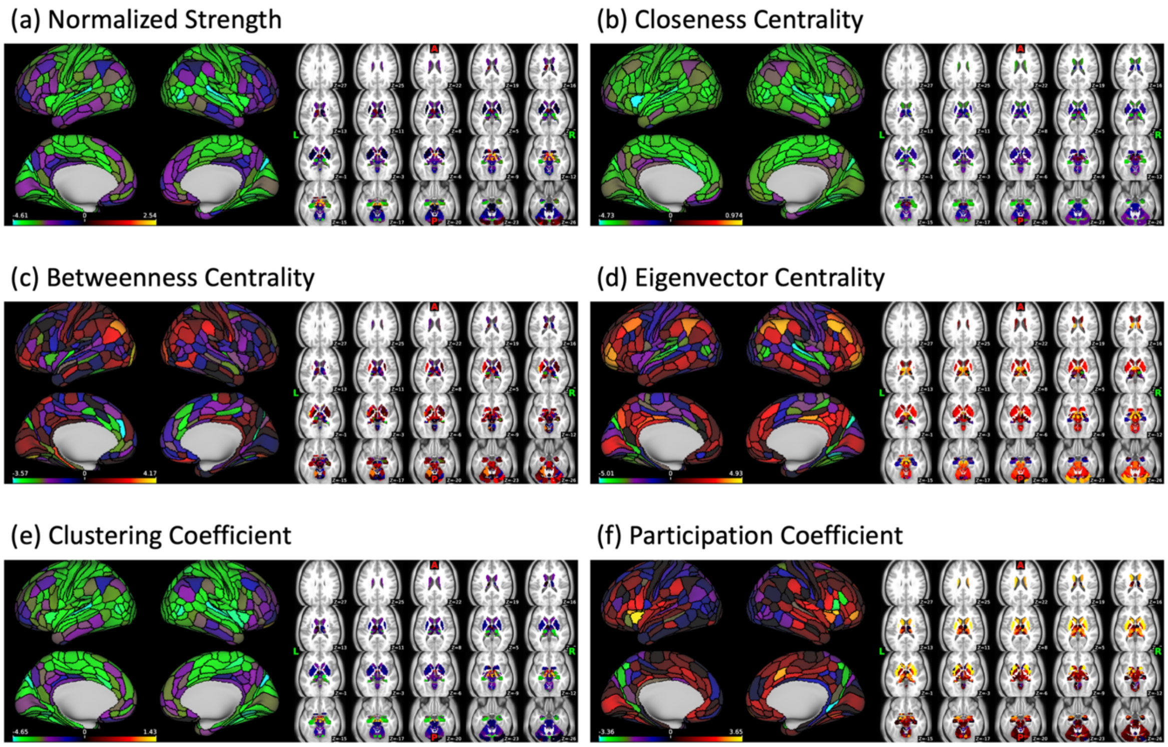

Supplementary Figure S2

Association of Age and Global Graph-Based Brain Functional Connectivity: Positive-Only Connectivity Weights. Modularity association with (a) age and (b) loneliness. Average shortest path length with (c) age and (d) loneliness.

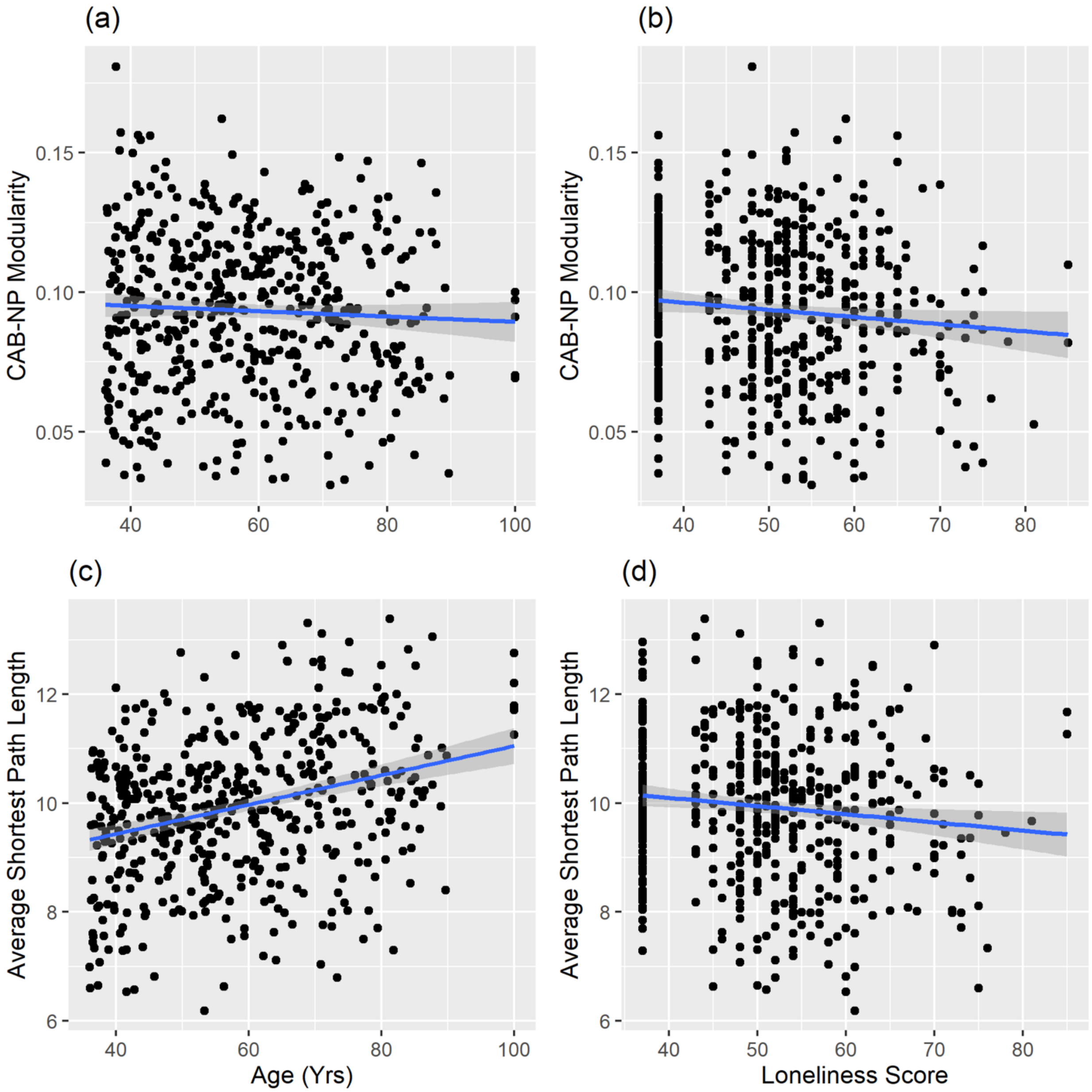

Supplementary Figure S3

Age and Graph-Based Brain Functional Connectivity Association Maps: Negative-Only Connectivity Weights. Color bar shows the z-score from Aspin-Welch's test for using exchangeability blocks to adjusted for four scanning sites and variance was estimated for each block, implemented in FSL PALM.

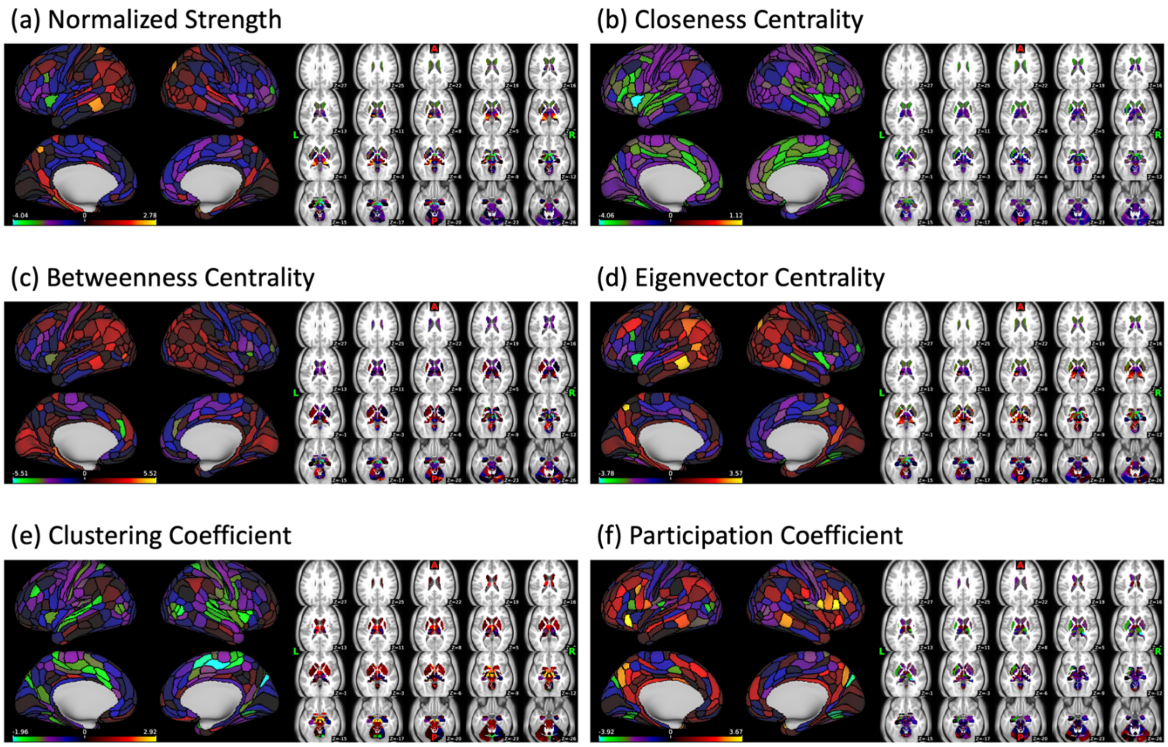

Supplementary Figure S4

Association of Age and Global Graph-Based Brain Functional Connectivity: Negative-Only Connectivity Weights. Modularity association with (a) age and (b) loneliness. Average shortest path length with (c) age and (d) loneliness.

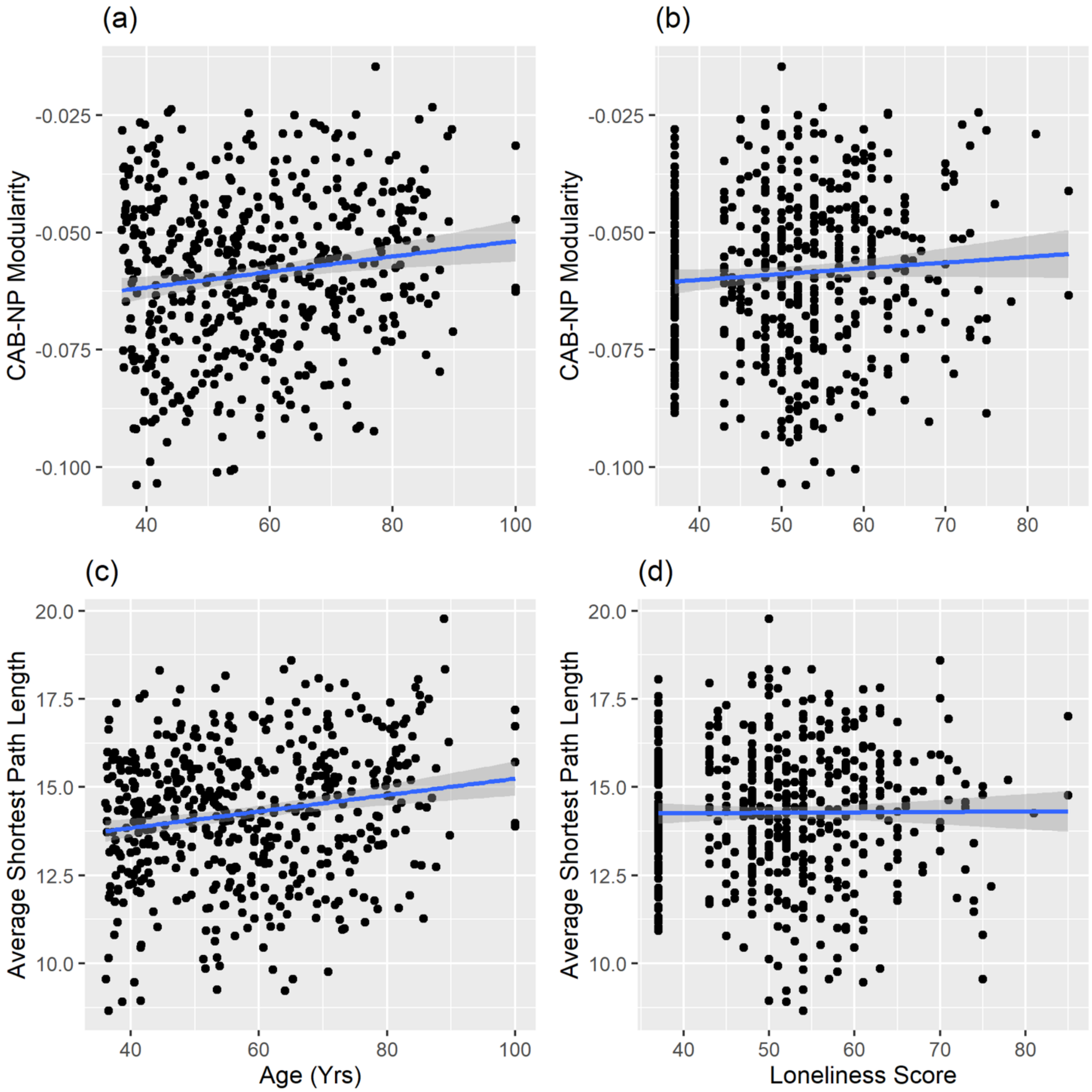

Supplement: Supplement 1 [file media-1.pdf]
